# Exploring Hierarchical Auditory Representation *via* a Neural Encoding Model

**DOI:** 10.3389/fnins.2022.843988

**Published:** 2022-03-24

**Authors:** Liting Wang, Huan Liu, Xin Zhang, Shijie Zhao, Lei Guo, Junwei Han, Xintao Hu

**Affiliations:** ^1^School of Automation, Northwestern Polytechnical University, Xi’an, China; ^2^Institute of Medical Research, Northwestern Polytechnical University, Xi’an, China

**Keywords:** hierarchical auditory representation, deep convolutional auto-encoder, naturalistic experience, neural encoding, fMRI

## Abstract

By integrating hierarchical feature modeling of auditory information using deep neural networks (DNNs), recent functional magnetic resonance imaging (fMRI) encoding studies have revealed the hierarchical neural auditory representation in the superior temporal gyrus (STG). Most of these studies adopted supervised DNNs (e.g., for audio classification) to derive the hierarchical feature representation of external auditory stimuli. One possible limitation is that the extracted features could be biased toward discriminative features while ignoring general attributes shared by auditory information in multiple categories. Consequently, the hierarchy of neural acoustic processing revealed by the encoding model might be biased toward classification. In this study, we explored the hierarchical neural auditory representation *via* an fMRI encoding framework in which an unsupervised deep convolutional auto-encoder (DCAE) model was adopted to derive the hierarchical feature representations of the stimuli (naturalistic auditory excerpts in different categories) in fMRI acquisition. The experimental results showed that the neural representation of hierarchical auditory features is not limited to previously reported STG, but also involves the bilateral insula, ventral visual cortex, and thalamus. The current study may provide complementary evidence to understand the hierarchical auditory processing in the human brain.

## Introduction

There are growing evidences supporting the hierarchy of auditory representations during auditory processing in the human brain (Chevillet et al., 2011; Sharpee et al., 2011; Durschmid et al., 2016; De Heer et al., 2017; Kell et al., 2018). For example, the neural processing of narrative speech involves hierarchical representations starting from the primary auditory areas and laterally to the temporal lobe (De Heer et al., 2017). In addition, the localization and identification of relevant auditory objects are accomplished *via* parallel “where” and “what” pathways (Ahveninen et al., 2006; Lomber and Malhotra, 2008; Bizley and Cohen, 2013). The hierarchy of neural auditory representation is important to understand what sensory information is processed as one traverses the sensory pathways from the primary sensory areas to higher-order areas.

In light of their hierarchical feature representation ability, recently advanced deep neural networks (DNNs) have gained increasing interest in exploring the hierarchy of neural auditory representation. These studies offer promising prospects to understand the fundamental mechanisms of brain functions responding to external stimuli. Specifically, brain encoding models (Naselaris et al., 2011; Han et al., 2014; Mesgarani et al., 2014; Du et al., 2019) have been used to establish the relationship between acoustic features represented in different layers of DNNs and brain activities. Brain regions of interest that selectively respond to extracted features in different layers were then inferred according to encoding performance. Using such a neural encoding framework, researchers have revealed a representational gradient in the superior temporal gyrus (STG) during auditory information processing (Evans and Davis, 2015; Kell et al., 2018; O’Sullivan et al., 2019; Kiremitçi et al., 2021). For example, Kell et al. (2018) found that latent features in intermediate network layers best predicted neural responses in the primary auditory cortex, while features in deeper layers can better explain brain activities in anterior, lateral and posterior directions of the non-primary areas.

In the majority of existing studies, the hierarchical features of external acoustic stimuli were derived using supervised DNNs that are designed for specific tasks, such as audio genre classification (Güçlü et al., 2016) or speech recognition (Kell et al., 2018). One possible limitation is that the supervised hierarchical representations could be biased toward discriminative features while ignoring the common ones shared by auditory excerpts in different categories. Consequently, the hierarchical organization of neural auditory processing revealed by the encoding model may be confined to classification or recognition domain. However, the neural processing of auditory information during naturalistic experience is not restricted to classification or recognition (Hasson and Honey, 2012; Fasano et al., 2020). Unlike supervised DNNs that use predefined labels as targets for model optimization, unsupervised DNNs such as deep convolutional auto-encoder (DCAE) adopts data reconstruction errors as objective functions and hence learn intrinsic and hierarchical features of input data directly (Masci et al., 2011). Thus, unsupervised DNNs may serve as possible tools to comprehensively map the hierarchy of neural auditory processing.

In this manuscript, we proposed an fMRI encoding framework to explore the hierarchy of neural auditory processing in the human brain. In brief, an unsupervised DCAE model (Masci et al., 2011), instead of supervised DNNs used in existing studies (Güçlü et al., 2016; Kell et al., 2018), was trained to derive unbiased hierarchical feature representations of naturalistic auditory excerpts in three semantic categories (pop music, classic music, and speech). An encoding model based on LASSO algorithm (Tibshirani, 2011) was learned to predict fMRI brain activities using acoustic features represented in each layer of the DCAE model. Brain regions that selectively response to the hierarchical features were inferred according to the encoding performance subsequently.

## Materials and Methods

### Overview

**FIGURE 1:**
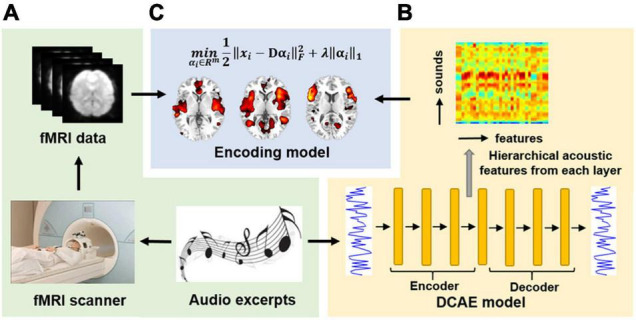
The schematic illustration of the study. **(A)** fMRI acquisition using naturalistic auditory excerpts as stimuli. **(B)** Hierarchical feature representation of the naturalistic auditory stimuli *via* an unsupervised DCAE model. **(C)** Hierarchical acoustic features were correlated to fMRI brain activities using an encoding model based on LASSO to infer hierarchical auditory representation in the brain.

As illustrated in Figure, we acquired fMRI data when the participants were freely listening to naturalistic auditory excerpts (Figure 1A). Then the hierarchical feature representations of each audio excerpt were derived *via* an unsupervised DCAE model (Masci et al., 2011; Figure 1B). Afterward, the hierarchical acoustic features were correlated to fMRI brain activities using an encoding model based on LASSO algorithm (Tibshirani, 2011; Figure 1C and Section “Encoding Model and Group-Wise Analysis”). In brief, the hierarchical feature representation was used to predict fMRI brain activities with a sparsity regularization, and the prediction accuracies was used to measure how well the acoustic features and brain activities were correlated. After that, a group-wise analysis was performed to identify brain regions whose activities were predicted with accuracies significantly above chance to infer hierarchical auditory representation in the brain.

### Functional Magnetic Resonance Imaging Acquisition and Preprocessing

Auditory excerpts in three semantic categories (classical music, pop music, and speech) were used as naturalistic stimuli in fMRI data acquisition. Each category was composed of seven excerpts and each excerpt was around 90 s. All excerpts were taken from legal copies of compressed MP3 audio files. These audio excerpts were aggregated in a random order to avoid the influence of the internal structure of audio data on human brain’s perception. FMRI data were acquired using a GE 3T Signa MRI system (GE Healthcare, Milwaukee, WI, United States) with an 8-channel head coil at the Bio-Imaging Research Center of the University of Georgia (UGA) under UGA Institutional Review Board (IRB) approval. Six healthy university students voluntarily participated in the study. The audio stimuli were delivered to the participants using an MRI-compatible audio headphone (Nordic NeuroLab, Bergen, Norway).

The detailed fMRI acquisition parameters were as follows: TR = 1.5 s, TE = 25 ms, 64 × 64 matrix, 30 axis slices, 4 mm slice thickness, 220 mm Field of View (FOV). FMRI data were pre-processed using FSL FEAT (FMRI Expert Analysis Tool) (Smith et al., 2004). The preprocessing included brain skull removal, slice timing and motion correction, spatial smoothing with 5 mm full-width at half-maximum (FWHM) Gaussian kernel, high pass temporal filtering, and linear registration to the standard Montreal Neurological Institute (MNI) brain template. After preprocessing, the time course of each voxel was normalized to have zero mean and unit standard deviation.

### Hierarchical Feature Representation Based on Deep Convolutional Auto-Encoder

#### Deep Convolutional Auto-Encoder Model

**FIGURE 2:**
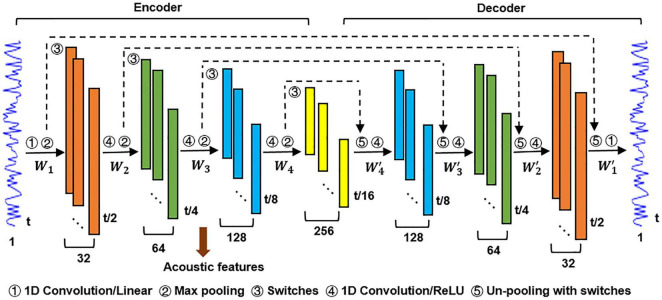
The DCAE model used for the hierarchical feature modeling.

The DCAE model used in this study is composed of an encoding block and a decoding block, as shown in Figure 2. The encoder transforms the input data into a detailed feature representation (feature maps), and the decoder performs data reconstruction (Masci et al., 2011). The objective of the DCAE model is to minimize the reconstruction errors between the input auditory signals and reconstructed ones.

Each block in the encoder consists of a convolutional layer and a max-pooling layer. A convolutional layer acts as feature extractor and the max-pooling layer reduces computational cost in the upper convolutional layer and gains translation/scale-invariance (Peterson et al., 2018; Song et al., 2018). Each block in the decoder consists of a deconvolution layer and an un-pooling layer. It is notable that the max-pooling operation is not invertible. To address this problem, we adopted a switch-based un-pooling approach (Zeiler and Fergus, 2014). The “switches” record the exact location of the max value in each pooling region during max-pooling, and then these “switches” are placed to its original position with corresponding max values (Huang et al., 2017). A linear activation function was applied in the first convolutional layer in the encoder and the last deconvolution layer in the decoder. The Rectified Linear Unit (ReLU) (Dahl et al., 2013) was used as activation function elsewhere. The objective function of the DCAE model consists of two terms. The first term represents the reconstruction error. The second term is an L2 regularization applied on weights to prevent overfitting and make the learned features more interpretable (Bilgic et al., 2014).

The number of layers in the DCAE model here was empirically set to balance the effectiveness of hierarchical feature learning and the interpretability of the subsequent inference of the hierarchical neural auditory processing. Intuitively, a larger number of layers would result in a finer featural representation of the input auditory excerpts. However, this would bring difficulties in interpreting the cortical hierarchy of acoustic feature processing in the human brain. In contrast, a smaller number of layers may not sufficient to learn the hierarchical feature representations of the input acoustic excerpts and consequently interrupt the encoding inference.

#### Deep Convolutional Auto-Encoder Parameter Settings and Model Training

During model training, the length of an input training sample was the same as the TR (1.5 s) in fMRI acquisition. The naturalistic auditory stimuli used in fMRI acquisition contribute 1,260 samples, which are not sufficient to train the DCAE model. To address this problem, we constructed additional 36,000 samples from the MagnaTagATune Dataset (Law et al., 2009) and the LibriSpeech Corpus (Panayotov et al., 2015) to pre-train the model (Data 1). The pre-trained model was then fine-tuned using the samples from the fMRI stimuli (Data 2). We implemented the DCAE model using Keras (Chollet, 2015) with CUDA and cuDNN. Based on our prior experiences (Huang et al., 2017), hyper-parameters in the DCAE including the number and the length of the filters were detailed in Table 1. The regularization parameter κ is experimentally set as 0.001. We used the Adam optimizer with default parameters β_1_ = 0.5, β_2_ = 0.999, epsilon = 1e^–8^ and a mini-batch size of 32 to train the model. We manually tuned the learning rate α = 0.0002 and weight decay = 0.001 to iteratively minimize the mean square error (MSE) loss function. The DCAE model converged after about 5,000 epochs.

**TABLE 1:** The number and length of filters in the DCAE model.

Filter number/filter length	Layer 1	Layer 2	Layer 3	Layer 4
Encoder	32/64	64/32	128/16	256/8
Decoder	256/8	128/16	64/32	32/64

#### Hierarchical Acoustic Feature Representation

Similar to a previous study (Kell et al., 2018), the acoustic features encoded in each of the four max-pooling layers in the encoder were regarded as a single level of the hierarchical feature representation of an input auditory sample. For each input sample (1.5 s*16k/s = 24k*1), its hierarchical feature maps on the four max-pooling layers are in the dimension of *t*_i_*c_*i*_, where *t*_*i*_ is the length of sample in the output of *i*-th max-pooling layer (24k, 12k, 6k, and 3k for *i* = 1, …, 4, respectively). *c*_*i*_ is the number of filters (channels) in the *i*-th convolutional layer. Following the feature dimensionality reduction strategy used in Güçlü et al. (2016), the high dimensional feature map on each max-pooling layer was temporally averaged, resulted in a *c*_*i*_-dimensional feature vector. For a given auditory excerpt consisting of 60 samples that was used as stimulus in fMRI acquisition, its hierarchical feature representation is in the dimension of 60*c_*i*_. Subsequently, each column of these hierarchical acoustic features was convolved with the canonical double-gamma hemodynamic response function (HRF).

### Encoding Model and Group-Wise Analysis

Linear encoding models are preferred in fMRI encoding studies due to their good interpretability (Naselaris et al., 2011). Compared to other linear regression models such as ridge regression and support vector regression (SVR) with a linear kernel, LASSO enforces a sparse encoding model that is able to identify a more compact set of variables of interest. Thus, an encoding model based on LASSO algorithm (Tibshirani, 2011) was trained to predict fMRI responses using the hierarchical feature representation described above. In the encoding model, we treated each 60-s auditory excerpt in fMRI acquisition and the corresponding individual excerpt-specific fMRI data as a single sample, resulting in a collection of 126 (3 auditory categories × 7 excerpts in each category × 6 participants) samples. The encoding model can be formulated as a matrix factorization with a sparsity penalty:


(1) minαi∈R12m||xi-Dαi||22+λ||αi||1

where *x*_*i*_ is the fMRI signal of each voxel in an individual participant, **D** is the corresponding hierarchical feature representation in each layer, *a*_*i*_ is the encoding coefficients, and λ is a sparsity controlling parameter. The encoding model was trained for each voxel independently. The encoding performance for each voxel was calculated as the Pearson correlation coefficient (PCC) between the predicted fMRI activities and the recorded ones. Repeating encoding model training and performance evaluating for each voxel resulted in an encoding performance map for each sample. The parameter λ balances the regression residual and sparsity level. The encoding model with a smaller λ better predicts *x*_*i*_ using a larger subset of **D** at the risk of over-fitting, while a larger λ decreases the prediction accuracy using a more compact subset of features. In our study, λ was varied from 0.05 to 0.15 with interval of 0.05 and was optimized *via* a leave-one-out cross-validation strategy to maximize the average encoding performance in the testing set.

A group-wise analysis was then performed to infer the corresponding brain regions that selectively encoded each level of the hierarchical feature representations in the DCAE model. In brief, for a given level of the hierarchical feature representations, the encoding performance map for each sample was independently normalized and aggregated to perform one-sample *t*-test to infer the corresponding brain regions that have encoding accuracy significantly above chance (*p* < 0.01, Z ≥ 2.3).

### A Comparison Study

**FIGURE 3:**
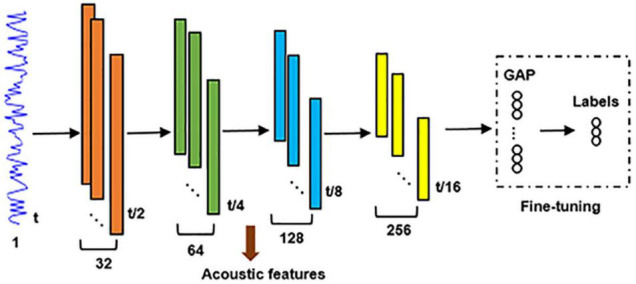
The architecture of the supervised DNNs for audio classification. GAP, global average pooling.

A comparison study was performed to compare the neural encoding of unsupervised hierarchical feature representations with that of a supervised classification model described as follows. A global average pooling (GAP) layer (Yu et al., 2017) followed by a fully connected soft-max classification layer were connected to the fourth max-pooling layer of the unsupervised DCAE model (Figure 3). Adopting cross-entropy loss function, Adam optimizer, early stopping strategy and batch size of 32, it was pre-trained using Data 1 and followed by fine-tuning using Data 2. Supervised hierarchical feature representations of input auditory excerpts were derived from the converged classification model. The neural encoding of supervised hierarchical features was probed using the same encoding framework described in Section “Encoding Model and Group-Wise Analysis” and was compared with that of the unsupervised DCAE model.

## Results

### Evaluation of Hierarchical Feature Learning

**FIGURE 4:**
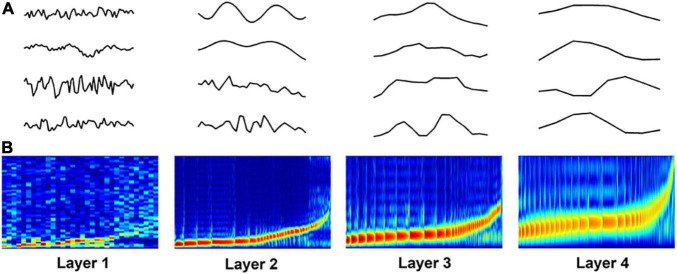
Visualization of learned filters in the DCAE model. **(A)** Examples of the learned filters in each layer. **(B)** The power-spectrum patterns of learned filters. The *x*-axis represents the index of filters, the *y*-axis represents the frequency ranging from 0 to 8000 Hz.

Figure 4A shows some examples of the learned filters in the DCAE model. The power-spectrum patterns of the learned filters are depicted in Figure 4B, where the filters in each layer are sorted according to the frequency (low to high) at which its magnitude reaches the maximum (Lee et al., 2018). In the first layer, the frequency of the filters increases approximately linearly in low frequency filter banks whereas filters that are selective for higher frequency are more spread out. As the layer goes deeper, the trend of frequency becomes non-linearly steeper in high frequency filter banks. These spectrum patterns are consistent with those in frame-level end-to-end learning for music classification (Dieleman and Schrauwen, 2014; Lee et al., 2018), suggesting the effectiveness of hierarchical feature learning in the DCAE model.

**FIGURE 5:**
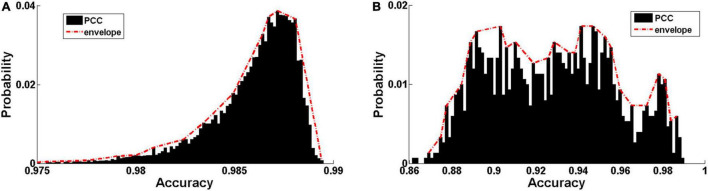
Encoding performance of the trained DCAE model. **(A)** The distribution of Pearson correlation coefficients (PCC) between the input audio signals and reconstructed ones in the MagnaTagATune dataset and LibriSpeech Corpus. **(B)** The distribution of PCC in the auditory samples from the fMRI stimuli.

The distribution of Pearson correlation coefficients (PCCs) between the input audio signals and reconstructed ones is shown in Figure 5. The PCC is relatively high in both Data 1 (0.9859 ± 0.0024, Figure 5A) and Data 2 (0.9274 ± 0.0297, Figure 5B). The discriminative ability of the hierarchical features learned by the DCAE model was then examined using a classification task based on support vector machine (SVM) with an RBF kernel. The classification performance in 5-fold cross-validations is summarized in Table 2 for each layer. The classification accuracy slowly increases as the layer goes deeper. Both the high data reconstruction performance and high classification accuracy indicate that the trained DCAE model could well capture the intrinsic features of the input samples. Similar classification results are observed in the supervised model (Table 3).

**TABLE 2:** The classification accuracies in different layers of the DCAE model (mean ± std).

	Layer 1	Layer 2	Layer 3	Layer 4
Data 1	0.7787 ± 0.0326	0.9079 ± 0.0060	0.9168 ± 0.0096	0.9198 ± 0.0077
Data 2	0.7528 ± 0.0221	0.9044 ± 0.0104	0.9084 ± 0.0169	0.9181 ± 0.0220

**TABLE 3:** The classification accuracies in different layers of the supervised model.

	Layer 1	Layer 2	Layer 3	Layer 4
Data 1	0.9093 ± 0.0160	0.9489 ± 0.0110	0.9558 ± 0.0086	0.9679 ± 0.0025
Data 2	0.8545 ± 0.1661	0.9309 ± 0.0139	0.9531 ± 0.0056	0.9552 ± 0.0033

### Encoding Performance

**FIGURE 6:**
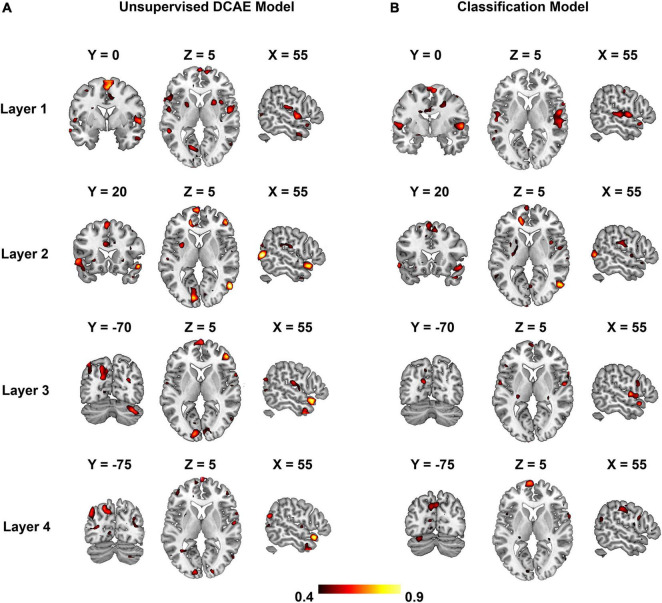
The encoding performance for each layer in the unsupervised DCAE model **(A)** and supervised classification model **(B)**.

The optimal sparsity controlling parameter λ = 0.1 maximized the overall encoding performance depicted in Figure 6 for the unsupervised DCAE (Figure 6A) and supervised classification model (Figure 6B). Each subgraph shows the PCC between the original fMRI signals and the ones predicted by the hierarchical feature representations in each layer. In general, the distribution of brain regions in each layer is similar in the unsupervised DCAE and supervised classification model. The primary auditory cortex is selective to acoustic features learned in the first layer, the non-primary auditory cortex in the superior temporal gyrus (STG) is more sensitive to intermediate-layer acoustic features, while the prefrontal cortex, visual cortex, and precuneus are involved in the processing of higher-level features learned in the last layer.

**FIGURE 7:**
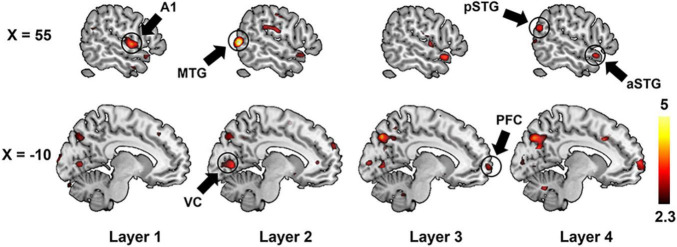
The comparison of encoding performance between the unsupervised DCAE model and supervised classification model in each layer. A1, primary auditory cortex; aSTG, anterior superior temporal gyrus; pSTG, posterior superior temporal gyrus; MTG, middle temporal gyrus; VC, visual cortex; PFC, prefrontal cortex.

We further adopted a paired-sample *t*-test to compare the encoding performance between the unsupervised DCAE and supervised classification models. It is observed that the encoding performance in some brain regions in the unsupervised DCAE model is significantly higher (*p* ≤ 0.01, Z ≥ 2.3) than those in the supervised classification model, including the primary auditory cortex (A1) in the first layer, part of middle temporal gyrus (MTG) and visual cortex in the second layer, anterior STG, posterior STG, part of prefrontal cortex (PFC), cuneus and precuneus in the third and last layer (Figure 7). No obvious brain regions showed significantly higher encoding performance in supervised classification model compared to the unsupervised one. These results suggested that the hierarchical features learned in the unsupervised DCAE model can achieve better encoding performance.

### Hierarchical Neural Auditory Representation

**FIGURE 8:**
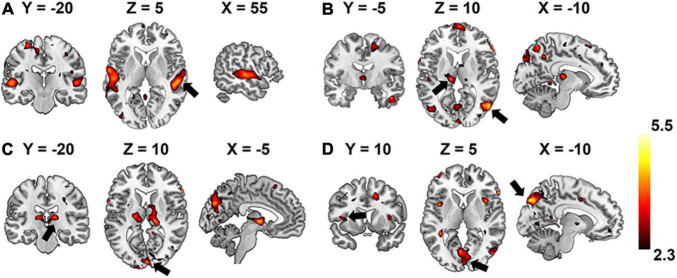
Brain regions that are selectively activated by the hierarchical acoustic features represented in each encoder layer of the unsupervised DCAE model. Panels **(A–D)** represent the first four layers in the unsupervised DCAE model.

Group-wise analysis was used to evaluate whether the encoding performance was significantly above chance (Z ≥ 2.3) for each voxel independently. Brain regions of interest that were selective to each level of the hierarchical acoustic feature representation were inferred accordingly to probe the hierarchy of neural auditory processing. Figure 8 shows the Z-maps of encoding performance for each encoder layer in the unsupervised DCAE model. In the first layer (Figure 8A), brain activities in the primary and association auditory cortices were with significantly (Z ≥ 2.3) high encoding accuracy, indicating that the features learned in the first layer may represent basic acoustic features. Part of the middle temporal gyrus (MTG) was activated in the second and third layer (Figures 8B,C). In the fourth layer, bilateral insula and ventral visual cortex were with significantly high encoding accuracy (Figure 8D). In addition, we observed that the thalamus was activated by the features represented in the second and third layers. The statistical details of these brain regions are listed in Supplementary Table 1.

**FIGURE 9:**
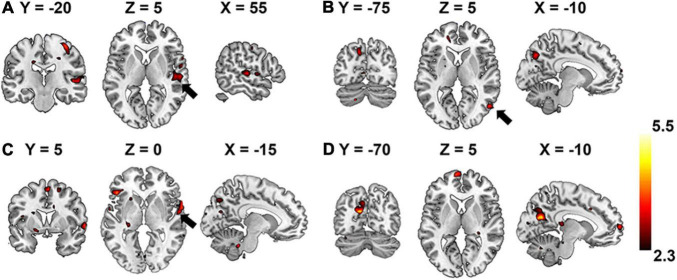
Brain regions that are selectively activated by the hierarchical acoustic features represented in each layer of the supervised classification model. Panels **(A–D)** represent the first four layers in the supervised classification model.

In comparison, the hierarchy of neural auditory processing revealed by the encoding model using supervised feature learning model is partly in line with the one in the unsupervised model, as shown in Figure 9. For example, the primary auditory cortex and visual cortex were selectively activated by the features represented in the first and fourth layer of the supervised model, respectively. However, those selective brain regions were much sparser and scattered distributed compared to the ones in the unsupervised model. In addition, the bilateral insula and thalamus were not activated in the supervised classification model.

## Discussion

In this study, we investigated the hierarchy of neural acoustic processing in the human brain *via* an fMRI encoding model. Compared to existing studies that used supervised feature learning models that are designed for classification or recognition to achieve a hierarchical feature representation of input acoustic information (Kell et al., 2018; O’Sullivan et al., 2019), the novelty of the current study is adopting an unsupervised DCAE feature learning model to derive intrinsic and unbiased hierarchical feature representation of naturalistic auditory stimuli in fMRI acquisition. Our experimental results showed that the neural representation of hierarchical auditory features is not limited to previously reported STG (Kell et al., 2018; O’Sullivan et al., 2019), but also involves the bilateral insula, ventral visual cortex and thalamus.

In the current study, our experimental results showed that the visual cortex and insula are related to the encoding of high-level acoustic features represented in the deepest layers of the DCAE model. It may indicate that these high-level features carry higher-order attributes such as emotion (Gu et al., 2013) and visual imagery (Vetter et al., 2014) elicited by auditory excerpts. For example, an fMRI study that uses auditory stimulation to examine the activity in the early visual cortex suggested that the auditory input enables the visual system to predict incoming information and could confer a survival advantage (Vetter et al., 2014). It also has been reported that the higher-level abstract or categorical information of acoustic stimulation is fed down to early visual cortex (Cate et al., 2009; Vetter et al., 2014). In addition, we observed that the thalamus may encode middle-level features. It has been reported that the thalamus plays an important role in auditory processing (Schonwiesner et al., 2006), especially for sound source localization (Proctor and Konishi, 1997), and tones modulated by attention (Frith and Friston, 1996). Our findings, in conjunction with previous results on the visual and auditory cortical representations (King and Nelken, 2009; Khalighrazavi and Kriegeskorte, 2014; Cichy et al., 2016), suggest that the existence of multiple representational gradients that processes increasingly complex conceptual information as we have experienced the sensory hierarchy of the human brain.

In the comparison study, the supervised model achieved better classification performance compared to the unsupervised DCAE model (Tables 2, 3). However, the unsupervised DCAE model outperformed the supervised model in terms of encoding performance (Figures 8, 9). More importantly, the cortical hierarchy pattern inferred by the supervised model was much sparser and scattered distributed compared to the ones in the unsupervised model (Figures 6, 7). These observations indicate that the intrinsic and unbiased hierarchical features learned in the DCAE model may provide additional evidence to understand the cortical hierarchy in neural auditory processing compared to the features learned in the supervised model that were biased toward discriminative ones while ignoring general attributes shared by auditory information in multiple categories.

In summary, the findings in this study may provide complementary evidences to understand the hierarchical auditory processing in the human brain. The current study can be improved in several ways in the future. It is expected to validate the findings using larger-scale fMRI datasets that recruit more participants. In the current study, we adopted an unsupervised DCAE model to derive the hierarchical feature representations of the acoustic stimuli in fMRI acquisition. The architecture and some of the hyperparameters (e.g., the number of layers, the number and length of the filters) of the DCAE model were empirically set. Although this DCAE model was able to effectively learn the hierarchical feature representation of the input acoustic excerpts as indicated by the SVM-based classification tasks in our experiments, it could be optimized by automated machine learning technique such as neural architecture search neural architecture search (NAS) (Elsken et al., 2019). In addition, the recently advanced self-supervised learning models (Sermanet et al., 2018; Li et al., 2020) may serve as more efficient and ecological approaches to unsupervised acoustic feature learning, and thus could enrich our understanding of the cortical hierarchy of neural auditory processing in future studies.

## Data Availability Statement

The raw data supporting the conclusions of this article will be made available by the authors, without undue reservation.

## Ethics Statement

The studies involving human participants were reviewed and approved by the Bio-Imaging Research Center of the University of Georgia. The patients/participants provided their written informed consent to participate in this study.

## Author Contributions

LW and XH designed the study, analyzed the data, and wrote the manuscript. HL analyzed the data. XZ, SZ, LG, and JH participated in the revision, reading, and approval of the manuscript. All authors contributed to the article and approved the submitted version.

## Conflict of Interest

The authors declare that the research was conducted in the absence of any commercial or financial relationships that could be construed as a potential conflict of interest.

## Publisher’s Note

All claims expressed in this article are solely those of the authors and do not necessarily represent those of their affiliated organizations, or those of the publisher, the editors and the reviewers. Any product that may be evaluated in this article, or claim that may be made by its manufacturer, is not guaranteed or endorsed by the publisher.
