# Supplementary material for: Exploring Hierarchical Auditory Representation via a Neural Encoding Model
Source: Front Neurosci. 2022 Mar 24;16:843988. doi: 10.3389/fnins.2022.843988 (PMC8987159; doi:10.3389/fnins.2022.843988)
Supplement: Supplementary file 1 [file Table_1.docx]

Supplementary Material

**Supplementary Table 1.** The statistical details of brain regions. Each value represents the number of significant voxels in standard MNI152 $2mm$ space (Z≥2.3).

| Layer | Brain region (AAL template) | Unsupervised DCAE | Classification |
| --- | --- | --- | --- |
| 1 | 'Temporal_Sup_L' | 498 | 11 |
|  | 'Temporal_Sup_R' | 618 | 299 |
|  | 'Temporal_Mid_L' | 212 | 133 |
|  | 'Temporal_Mid_R' | 198 |  |
| 2 | 'Temporal_Sup_L' | 44 |  |
|  | 'Temporal_Sup_R' | 90 |  |
|  | 'Temporal_Mid_L' | 183 |  |
|  | 'Temporal_Mid_R' | 495 | 166 |
|  | 'Thalamus_L' | 183 |  |
|  | 'Thalamus_R' | 51 |  |
|  | 'Calcarine_L' | 120 |  |
|  | 'Calcarine_R' | 25 |  |
|  | 'Cuneus_L' | 160 |  |
|  | 'Cuneus_R' | 37 |  |
|  | 'Precuneus_L' | 345 | 132 |
|  | 'Precuneus_R' | 154 |  |
| 3 | 'Temporal_Sup_L' | 36 | 71 |
|  | 'Temporal_Sup_R' | 82 | 53 |
|  | 'Temporal_Mid_L' | 134 |  |
|  | 'Temporal_Mid_R' | 239 |  |
|  | 'Thalamus_L' | 220 |  |
|  | 'Thalamus_R' | 307 |  |
|  | 'Precuneus_L' | 335 | 101 |
|  | 'Precuneus_R' | 164 |  |
|  | 'Calcarine_L' | 155 |  |
|  | 'Calcarine_R' | 108 |  |
|  | 'Cuneus_L' | 361 |  |
|  | 'Cuneus_R' | 135 |  |
| 4 | 'Insula_L' | 145 |  |
|  | 'Insula_R' | 131 |  |
|  | 'Calcarine_L' | 409 | 47 |
|  | 'Calcarine_R' | 202 |  |
|  | 'Cuneus_L' | 658 | 236 |
|  | 'Cuneus_R' | 157 |  |
|  | 'Precuneus_L' | 665 | 287 |
|  | 'Precuneus_R' | 303 | 154 |
